# Assessing biological and technological variability in protein levels measured in pre-diagnostic plasma samples of women with breast cancer

**DOI:** 10.1186/s40364-017-0110-y

**Published:** 2017-10-17

**Authors:** Christine Y. Yeh, Ravali Adusumilli, Majlinda Kullolli, Parag Mallick, Esther M. John, Sharon J. Pitteri

**Affiliations:** 1 Department of Biomedical Informatics, Stanford University School of Medicine, Stanford, CA 93405 USA; 2 Department of Radiology, Canary Center at Stanford for Cancer Early Detection, Stanford University School of Medicine, Palo Alto, CA 94304 USA; 3 Department of Genetics, Stanford University School of Medicine, Stanford, CA 93405 USA; 4 Stanford Cancer Institute, Stanford University School of Medicine, Stanford, CA 94305 USA; 5 Cancer Prevention Institute of California, Fremont, CA 94538 USA

**Keywords:** Breast cancer, Protein, Mass spectrometry, Immunoassay, Blood plasma

## Abstract

**Background:**

Quantitative proteomics allows for the discovery and functional investigation of blood-based pre-diagnostic biomarkers for early cancer detection. However, a major limitation of proteomic investigations in biomarker studies remains the biological and technical variability in the analysis of complex clinical samples. Moreover, unlike ‘omics analogues such as genomics and transcriptomics, proteomics has yet to achieve reproducibility and long-term stability on a unified technological platform. Few studies have thoroughly investigated protein variability in pre-diagnostic samples of cancer patients across multiple platforms.

**Methods:**

We obtained ten blood plasma “case” samples collected up to 2 years prior to breast cancer diagnosis. Each case sample was paired with a matched control plasma from a full biological sister without breast cancer. We measured protein levels using both mass-spectrometry and antibody-based technologies to: (1) assess the technical considerations in different protein assays when analyzing limited clinical samples, and (2) evaluate the statistical power of potential diagnostic analytes.

**Results:**

Although we found inherent technical variation in the three assays used, we detected protein dependent biological signal from the limited samples. The three assay types yielded 32 proteins with statistically significantly (*p* < 1E-01) altered expression levels between cases and controls, with no proteins retaining statistical significance after false discovery correction.

**Conclusions:**

Technical, practical, and study design considerations are essential to maximize information obtained in limited pre-diagnostic samples of cancer patients. This study provides a framework that estimates biological effect sizes critical for consideration in designing studies for pre-diagnostic blood-based biomarker detection.

**Electronic supplementary material:**

The online version of this article (10.1186/s40364-017-0110-y) contains supplementary material, which is available to authorized users.

## Background

Early detection of breast cancer greatly increases prognosis with a 5-year survival rate of >98% for localized disease versus 29.6% survival for disease diagnosed at a metastatic stage [1]. Systematic blood-based studies using a variety of protein assay technologies have profiled hundreds of proteins and identified candidate targets that differentiate between cancer patients and matched controls [2, 3]. Only a small subset of these targets has been validated in additional samples [4]. One major limitation is that most of these discovery studies have relied on samples that were collected at the time of cancer diagnosis. Using such samples makes the key assumption that proteins indicative of disease pathology in cancer patients can be extrapolated to pre-diagnostic samples. Due to the relative scarcity of pre-diagnostic blood samples, few biomarker studies have used such samples, and they were primarily used in validation studies, often with negative results even with initially high-confidence protein targets [5]. Molecular profiles of blood in cancer progression have been shown to change in longitudinal studies [6], indicating that particular protein analytes are only predictive of disease outcomes within a limited lead time [4]. This henceforth reinforces the need to conduct exploratory studies in pre-diagnostic samples to identify early cancer diagnostic protein targets.

Despite valuable progress in the limited efforts to detect biomarkers in pre-diagnostic breast cancer samples [7–9], challenges of small samples sizes and technical/biological noise persist. Meta-analyses of the proteomic revolution and its application to cancer biomarker discovery highlights the amount of inter-individual variability that interferes with true analytical variability in clinical samples [9]. Some of the variation may be due to lifestyle factors [10], and twin studies measuring plasma proteins have emphasized the need to account for genetic, environmental, and temporal variability [11]. Moreover, variability in clinical samples may also arise from therapies received by cancer patients. Studies based on samples from patients using menopausal hormone therapy [12–14] have highlighted the need for cancer biomarker studies to consider the confounding effect of hormone therapy on protein levels.

Given the known variability in protein expression in the blood, the implications of technical and practical choices for biomarker discovery on often limited numbers of samples need to be considered. Immuno-based methods once dominated the field, and are still commonly used in biomarker studies [15]. However, technical advancements in mass spectrometry now allow for shotgun proteomics that provide unbiased relative protein measurements with higher coverage. Emerging reaction monitoring [16], and sequential window acquisition of all theoretical mass spectra [17] mass spectrometry-based technologies have also started to build quantitative assays for larger numbers of proteins. Each type of platform comes with advantages, disadvantages, and varying ability to detect true analytical signals beyond the biological noise. We thus present, to our knowledge, the most extensive characterization of pre-diagnostic samples of ten breast cancer cases with side-by-side comparison of select proteomics platforms with the goals of characterizing technical and biological variation.

## Methods

### Human plasma samples

The blood plasma samples used in this study were obtained from the Northern California site of the Breast Cancer Family Registry (BCFR) [18]. Women with newly diagnosed breast cancer (probands) were enrolled in the family registry from 1996 to 2011, as well as their sisters and other relatives, and followed prospectively to 2017. At enrollment, probands completed a cancer family history questionnaire and all participants completed a questionnaire on epidemiologic risk factors for breast cancer, including personal history of cancer, and provided a blood sample that was stored in -70C freezers for future research. Other health conditions were not assessed at enrollment. Probands were interviewed annually and asked about new cancer diagnoses among family members. More extensive follow-up questionnaires were administered to probands and relatives in 2012–2014 and 2015–2017. For this pilot study, we selected stored plasma samples from 10 sister pairs from women who did not have a personal history of breast cancer when they enrolled in the family registry. They included 10 women who were diagnosed with breast cancer within 24 months of providing the blood sample and 10 of their biological full sisters who did not develop breast cancer within 24 months of providing the blood sample.

Case samples. The ten women with breast cancer were diagnosed between 1997 and 2007 and nine diagnoses were confirmed by pathology reports, medical records, or the cancer registry. One diagnosis was based on a proband report only, as the affected individual did not participate in follow-up. None of these 10 women had a cancer diagnosis before enrollment in the family registry.

Control samples. Nine of the 10 control women completed follow-up questionnaires between 2012 and 2017 and did not report any cancer diagnosis. Three of the control women were California residents and linkage with the California Cancer Registry in 2013 did not reveal any cancer diagnoses. One control woman had a cervical cancer diagnosis 10 years before enrollment and a breast cancer diagnosis 18 years after enrollment in the family registry; both diagnoses were confirmed by the cancer registry. (Additional file 1: Table S1).

### Sample preparation for shotgun proteomic analysis

**Fig. 1:**
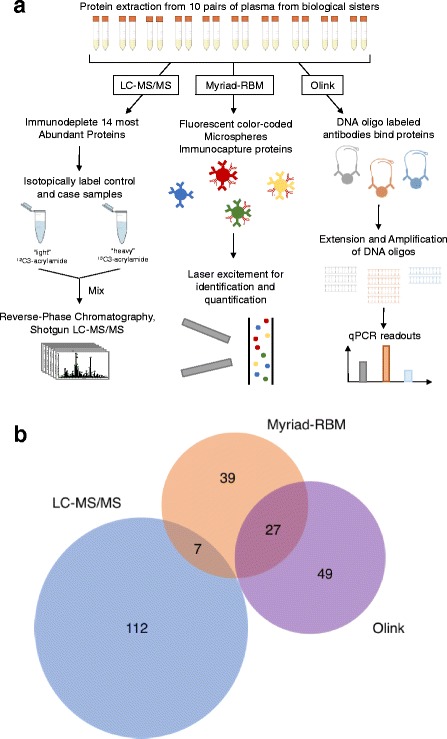
Overview and Summary of Study. **a** Study design and workflow. **b** Number of distinct proteins measured across the three platforms; LC-MS/MS, Myriad-RBM, and Olink

The plasma mass spectrometry-based proteomics workflow used in this study was adapted from a previously described workflow [13] (Fig. 1a). Briefly 250 μL aliquots of each plasma sample were subjected to immunodepletion. The fourteen most abundant proteins (including albumin, immunoglobulins G (IgG) and A (IgA), transferrin, haptoglobin, α-1-antitrypsin, fibrinogen, α-2-macroglobulin, apolipoprotein A1, and acid-1-glycoprotein) were removed from the plasma samples using CaptureSelect™ HumanPlasma14 depletion material [19]. The proteins in the depleted plasma samples were then isotopically labeled with the control samples receiving “light” ^12^C3-acrylamide and the case samples receiving “heavy” ^13^C3-acrylamide [20]. The case and control samples were mixed and fractionated by reversed phase chromatography using a C8 column (POROS R2). Fractions were then lyophilized and digested with trypsin in solution.

### LC-MS/MS protein quantification

The tryptic peptide samples were analyzed by liquid chromatography-tandem mass spectrometry (LC-MS/MS) on a LTQ-Orbitrap Velos mass spectrometer coupled with an Eksigent nanoLC. The resulting LC-MS/MS data were searched against the human UniProtKB database using the Computational Protein Analysis System from X!Tandem. Search results were analyzed by PeptideProphet [21] and peptides with a score > 0.75 were retained for protein identification and quantitation. Quantitative information from peptide signal intensities was then extracted using Q3ProteinParser, and ratios of heavy-to-light acrylamide-labeled peptides were computed for each protein in each of the ten sample pairs (Additional file 2) [20]. The estimated absolute protein abundance levels for the LC-MS/MS method were based on a reference database of 1200 canonical proteins [22].

### Myriad-RBM analysis

We used the Human OncologyMAP® v.1.0 assay (Luminex xMap technology, Myriad-RBM Inc., Austin TX) from Myriad-RBM Inc. to measure a targeted panel of 101 potential protein biomarkers such as cancer antigens, interleukins, and proteases (Additional file 1: Table S2) in the 20 samples. Two hundred microliters of each plasma sample was submitted for analysis. This assay uses color-coded microspheres with antibodies specific to each of the target protein to capture and detect specific analytes in each sample. Lasers then simultaneously excite the reporter dye that quantifies the analyte specific to each microsphere, and the internal fluorescent dye that identifies the microsphere (Fig. 1a). The Myriad-RBM analysis provides the measured concentration of each target analyte in a sample, the least detectable concentration (LDD), as well as the low to high normal range for all analytes (Additional file 2).

### Olink analysis

Protein levels of an additional 96 potential biomarkers (Additional file 1: Table S2) were analyzed in the 20 samples using the Olink Proseek Multiplex Oncology I 96 × 96 kit (Additional file 2) [23]. Twenty microliters of each plasma sample was submitted for analysis. For each target analyte in this assay, a pair of oligonucleotide-labelled antibodies probes bind to the protein in each sample. When the two probes are near each other after binding to the target protein, a PCR target sequence is formed by a proximity-dependent DNA polymerization event. The resulting sequence is subsequently detected and quantified using standard real-time quantitative polymerase chain reaction (qPCR) on the Fluidigm BioMark HD real-time PCR platform (Fig. 1a). All assay characteristics including detection limits and measurements of assay performance and validations are available from the manufacturer’s webpage (http://www.olink.com/products/oncology/).

### Data filtering

In the LC-MS/MS analysis, only proteins quantified in all ten case-control mixed samples were used in downstream quantitative analysis. For Myriad-RBM and Olink analyses, only analytes with at least 80% of valid measurements (defined as measurements above LDD for Myriad-RBM platform) across all samples were kept for downstream quantitative analyses. These cutoffs were made to retain statistical power in downstream quantitative analyses.

### Inter-assay comparisons

Inter-assay comparisons were visualized by standard boxplots and linear regression scatter plots. The adjusted R^2^ metric defined the strength of correlation between measurements of the same proteins across different platforms.

### Intra-assay technical variation

Protein assays have inherent variability known as technical variation across technical replicates. The statistical metric used to evaluate technical variation in the LC-MS/MS and Olink platforms was relative error (defined as the standard deviation divided by the mean across multiple replicates). The intra-assay technical variation of the LC-MS/MS method was evaluated by the density of relative error for case-to-control ratios; the relative error was calculated for all proteins using multiple peptides for the same protein as replicates. Meanwhile, for the Olink analysis we evaluated relative error for triplicate measurements for each protein analyte.

### Biological versus technical variance

For the Olink assay, technical variation was calculated as the mean of standard deviation across each protein analyte and biological variation was calculated as the standard deviation of mean measurements across each protein analyte. The variance was first analyzed by comparing biological variance with technical variance. Furthermore, to assess variance decomposition, we then averaged the type-II ANOVA sum of squares to evaluate the proportions of variance.

### Principal component analysis (PCA)

Principal Component Analysis, an unsupervised learning method, was used to reduce the multi-dimensional Myriad-RBM and Olink datasets into the first two orthogonal components that capture the major sources of variation.

### Student’s *t*-test and multiple hypothesis correction

Paired and unpaired *t*-tests with *p*-values corrected by the Benjamini-Hochberg multiple hypothesis testing were used to evaluate quantitative differences between case and control samples.

## Results

As shown in Fig. 1b, the LC-MS/MS method measured relative levels of 119 proteins, while the Myriad-RBM and Olink methods measured absolute levels of 73 and 76 proteins, respectively, for all samples. No proteins were measured by all three methods. Seven proteins were measured by both LC-MS/MS and Myriad-RBM, and 27 proteins were measured by both the Olink Biosciences and Rules Based Medicine assays. The three methods provided ample information to evaluate the variability of current protein assays in detecting protein biomarkers for early breast cancer diagnosis.

### Depth and frequency of protein measurements

**Fig. 2:**
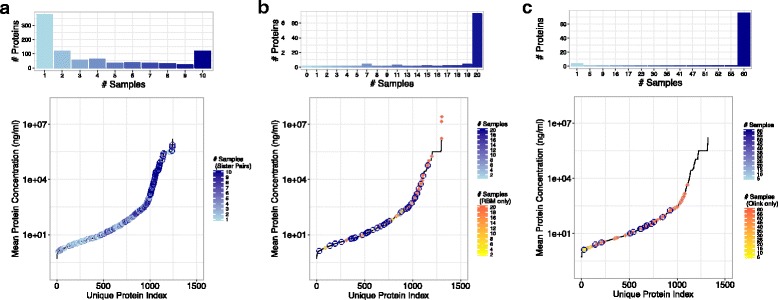
Depth of analyses and measurement frequency across platforms. Histogram (top) of frequency that a protein is quantified in *n* number of samples, and dynamic range plot (bottom) of mean concentrations measured for each unique protein quantified in platforms (**a**) LC-MS/MS, (**b**) Myriad-RBM, (**c**) Olink

The LC-MS/MS method provided relative quantitation values for proteins over six orders of magnitude (Fig. 2a). Similarly, Fig. 2b shows that the Myriad-RBM method also quantified proteins over six orders of magnitude, whereas the Olink method quantified proteins over a relatively smaller range of four orders of magnitude (Fig. 2c). Overall, the Olink platform captured lower abundance and smaller range of protein levels compared to the other two methods.

The frequency of unique protein measurements across plasma samples is plotted in the histograms of Fig. 2a-c for the three methods. Most proteins were measured in all 20 samples by the Myriad-RBM and Olink methods. Conversely, the LC-MS/MS method quantified the largest total number of proteins (1200), but also yielded missing data: only 13.5% of the total number of quantified proteins was measured in all ten pairs of “heavy” and “light” mixed samples. We chose to focus on only the 119 proteins identified across all samples in the LC-MS/MS method for downstream analysis.

### Assay-to-assay comparison in antibody-based technologies

**Fig. 3:**
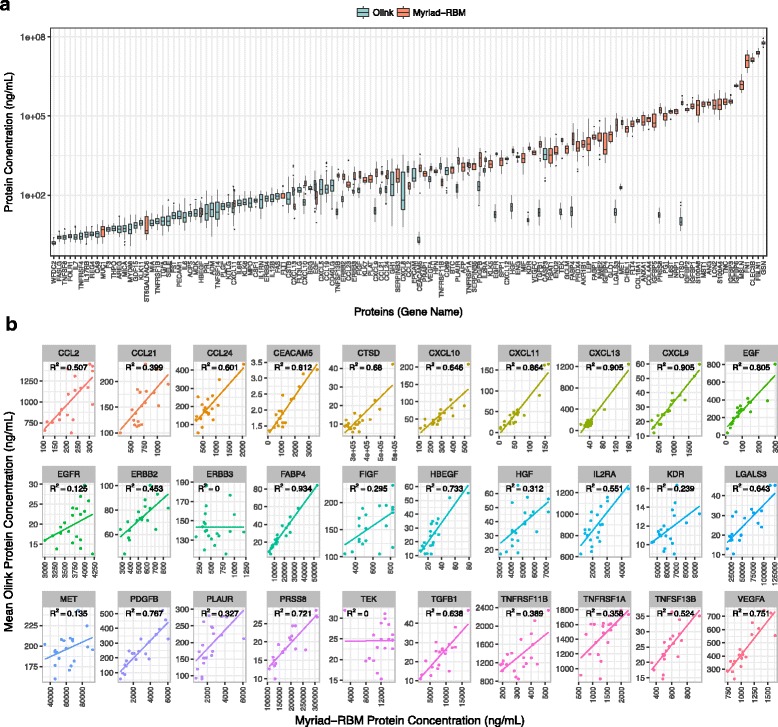
Assay-to-Assay Comparison for Antibody-Based Technologies. **a** Myriad-RBM estimates higher absolute protein concentration in comparison to Olink platform. **b** Protein levels are concordant for most proteins measured in both antibody-based platforms

The Myriad-RBM and Olink assays are both antibody-based technologies that aim to capture absolute concentrations of protein analytes in biological samples. We therefore compared the results from each of the two panels. Fig. 3a shows boxplots of protein measurements across all genes sorted by mean abundance for both methods. In general, the Myriad-RBM assay provided higher absolute level measurements than the Olink assay, even for analytes that were measured by both assays. This is particularly noteworthy as each method used absolute standards to determine values for protein concentrations.

Despite the discrepancy of absolute measurements between these two antibody-based assays, we saw that at least the measurements were mostly concordant for the 30 proteins measured by both methods (Fig. 3b), although the strength of correlation varied from protein analyte to analyte.

### Technical variation in LC-MS/MS and Olink analysis

**Fig. 4:**
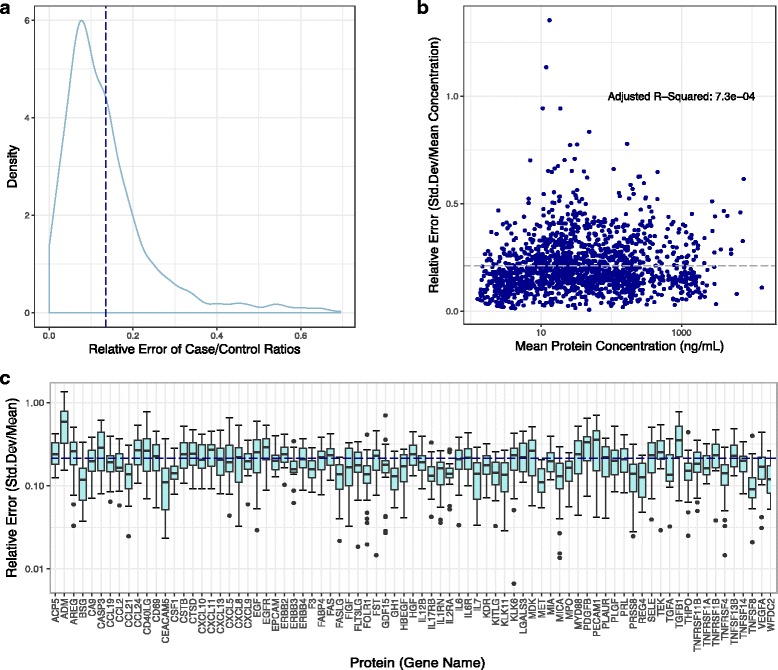
Technical Variation. **a** Relative error of case/control ratios across all peptides per proteins average at 10% (dashed line) in LC-MS/MS platform. **b** Relative error independent to protein abundance in Olink analysis determined by adjusted R^2^ value from linear regression. **c** Relative error across all proteins measured in the Olink analysis averages at 20% (dashed line)

Each assay has inherent technical variation. We were able to measure variation across technical replicates for the LC-MS/MS and Olink Analyses. We concluded that the LC-MS/MS method had a mean relative error for each measured peptide at approximately 10% for this dataset (Fig. 4a). The relative error across the triplicates for each analyte in the Olink analysis was plotted as boxplots in Fig. 4c. This figure highlights that the average relative error was found to be approximately 20% across all analytes, but the relative error was not consistent across analytes. Moreover, as shown in Fig. 4b, there was no evidence that relative error is correlated with measured protein abundance. Together, Fig. 4b-c suggest that inherent technical variation is specific to analyte and not necessarily specific to measurement platform or measured protein abundance.

### Biological versus technical variation

**Fig. 5:**
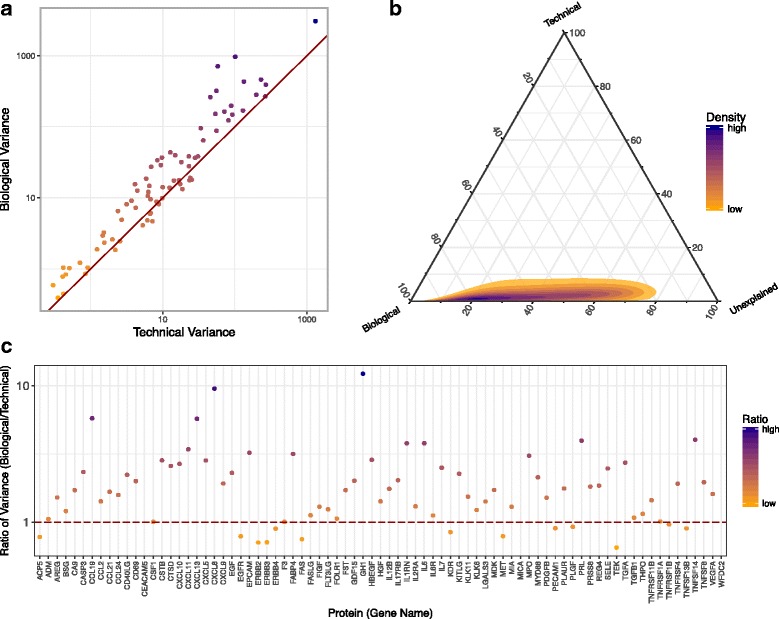
Biological versus Technical Variation. **a** Biological variance is generally higher than Technical Variance in the Olink Assay. **b** Variance decomposition shows that most variance can be explained by biological variability across the samples. **c** Each protein measured has its own ratio of biological variance vs. technical variance

Figure 5a shows a scatterplot of technical variation versus biological variation in the Olink assay. Each point represents a protein and points above the line indicate that the biological variation in the measurements was higher than the technical variation of the measured protein levels. A variance decomposition ternary plot, shown in Fig. 5b, exhibits that variance is mostly captured by variability between individuals rather than technical variance or random error. Though there was inherent technical variance in the Olink measurements as shown in Fig. 3, we were still able to detect biological signals across the individuals in the study.

Furthermore, it is noteworthy that both the biological and technical variations were specific to the protein analyte measured in the Olink Assay. The ratio of biological to technical variation for each protein was plotted in Fig. 5c. Some proteins show more biological variability than other proteins; it is expected that in plasma from breast cancer cases some proteins will behave similarly, whilst others would vary depending on personal variability. We observed that in the Olink analysis, variability was mostly bounded by biological variance, although a deeper look revealed that some individual protein analytes had inherently more individual personalized biological variability.

### Case to control comparison

An unsupervised principal component analysis of the Myriad-RBM and Olink datasets showed that the two principal components captured more than 90% of the variation in both the antibody-based assay datasets. However, there was no observable discrimination between the case and control samples in this reduced dimensional space (Additional file 3: Figure S1A).

**Fig. 6:**
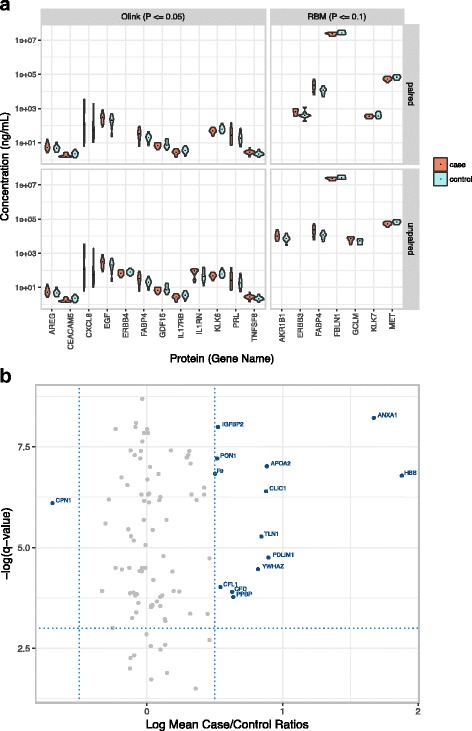
Case and Control Differences. **a** Unpaired and paired *t*-tests reveal few case/control ratios that are significant in the targeted proteomics assays. **b** Volcano plots reveal protein analytes that characterize difference between case and controls (blue points) from shotgun LC-MS/MS measurements

Violin plots in Fig. 6a summarize the most significant proteins from unpaired and paired *t-*tests on measurements from the antibody-based assays. Only a few proteins showed differences with *p*-value less than 1E-01 in the Myriad-RBM analysis; and within the group of five significant analytes measured, we observed only minimal differences between the means of the cases and controls. We observed that the Olink platform identified more significant proteins (*p*-value <1E-01), with better separation of the case and control distributions. Fatty Acid Binding Protein 4 (FABP4), a protein highly expressed in the liver, was found to be significantly different between cases and controls from both Olink and Myriad-RBM measurements. Some significant analytes were mutually exclusive to the paired and unpaired analyses in the Myriad-RBM platform, but not in the Olink analysis.

Figure 6b shows ten proteins with both the largest absolute fold differences and most significant Benjamini-Hochberg corrected *p*-values from a parallel paired *t*-test analysis of the LC-MS/MS data. Amongst these top ten proteins, most are commonly observed in the blood but not previously explicitly linked to cancer-related molecular mechanisms.

## Discussion

We evaluated three different types of assays (Fig. 1a) currently used for protein biomarker studies for their respective figures of merit and practical considerations. We found that each platform delivered varying degrees of depth and range for each individual analyte measured (Fig. 2), even in the cases where the two immuno-based assays claim to measure the absolute values of the same protein (Fig. 3). We found that platforms had inherent technical variation evaluated with technical replicates (Fig. 4); however, we were still able to detect biological signal that generally surpassed technical variation (Fig. 5a, c). Despite that most variability was captured by biological effects (Fig. 5c), paired and unpaired comparisons of pre-diagnostic case and control samples yielded 32 significant proteins (Fig. 6) with altered expression levels, with a limited subset previously reported to be associated with cancer. No proteins, however, retained statistical significance after false discovery correction. We conclude that technical, practical, and study design considerations are essential to maximize information obtained in limited pre-diagnostic samples of cancer patients.

The motivation for this study was to perform an exploratory investigation of technical and study design implications for identifying potential protein biomarkers in a small sample (*n* = 10) of pre-diagnostic breast cancer biospecimens. The pre-diagnostic plasma samples from the BCFR in this study – and human clinical cancer biospecimens in general – are often relatively limited in number and amount and difficult to come by. Thus one often arrives, as we did in this study, at a situation wherein there are multiple available technologies to analyze precious samples. It is therefore critical to understand the implications of technical choices, and to carefully design experiments to maximize information obtained.

In this study, we selected three different types of assays (Fig. 1a) currently used for protein biomarker studies. We evaluated each type assay for their respective merit and practical considerations. We leveraged specimens from matched sisters who remained cancer-free as controls and compared them to pre-diagnostic specimens of sisters who developed breast cancer within 2 years of providing the blood sample. Therefore, in addition to comparatively assessing the technical platforms, we also evaluated whether a paired design would bring more statistical power to account for confounding biological variability. Though modest in sample size, this study serves as a model pilot study and practical strategy to evaluate important factors before designing a larger study.

Each of the three methods evaluated in this study comes with their own advantages and disadvantages. The unbiased shotgun LC-MS/MS method not only avoids a constrained pre-selection of analytes of interest, but at the same time can also quantify many more proteins than targeted panels such as the Myriad-RBM and Olink platforms (Fig. 2a). However, LC-MS/MS and most other quantitative mass spectrometry-based methods provide relative rather than absolute quantitation. Absolute measurements require the use of internal standards, which is not feasible in an untargeted study of hundreds of proteins. In recent years, emerging mass spectrometry-based technologies [16] have started to build quantitative assays for larger numbers of proteins; however, these technologies require either a priori knowledge of analytes of interest, or peptide spectral libraries that still rely on data dependent acquisition methods as exhibited in this study. The LC-MS/MS approach is also more sensitive towards higher abundance proteins, thus impacted by a relatively large amount of missing data compared to the antibody-based approaches due to the inherent under-sampling of the mass spectrometer.

In contrast to the LC-MS/MS method, the Myriad-RBM and Olink approaches provide absolute measurements of specific protein levels (Additional file 1: Table S2) which are useful for targeted rather than exploratory studies. These platforms provide respective datasets with protein measurements for nearly every analyte in every sample, with little missing data (Fig. 2b-c). However, though the subset of proteins that were measured by both platforms provided mostly concordant values (Fig. 3b), there remained a clear discrepancy in absolute measurements between the platforms (Fig. 3a) which raises concerns about the reliability of these reported absolute values. This discrepancy is possibly due to differential calibrations used by the different technologies. Moreover, for protein measurements that were not concordant between the two antibody-based methods, it is possible that corresponding antibodies may not be measuring the same epitope of a protein and are henceforth capturing different protein forms. Another disadvantage of these antibody-based technologies is that antibodies can either be inherently biased due to their affinity to substrates, or unavailable for potential novel protein biomarkers.

In addition to the technical features of these assays, there are several practical considerations for each approach. The LC-MS/MS approach was the most time-consuming approach due to time needed to perform fractionation to overcome the large dynamic range of proteins in the blood and the time required for LC-MS/MS data acquisition. The Olink assays required the smallest volume of plasma samples of all the platforms, which is an important practical consideration when working with the precious and limited pre-diagnostic samples. Lastly, each assay comes at a different cost which may determine the feasibility of performing assays in duplicate, or even triplicate.

The three assay platforms provide both complementary and mutually exclusive information. Therefore, practical considerations and priorities may determine the choice of assay for evaluating clinical samples for biomarker discovery. All assays used in this this study had some inherent technical variation. We also chose to evaluate technical variation of the antibody-based Olink platform rather than the Myriad-RBM platform largely because the Olink assay requires significantly smaller volume of our limited biospecimens. We found some technical variation in the Olink assay, but it was not correlated with the type of analyte or measured protein abundance (Fig. 4b-c), thus establishing reliable scalability of the assay across all protein targets.

Despite technical variation detected with our triplicate analysis, we observed that the signal from biological variation of protein levels between samples was larger than the technical variation (Fig. 5a-b). Interestingly, each protein analyte in the same antibody-based platform has its own technical and biological variation (Fig. 5c), which cannot necessarily be predicted a priori, probably owing to inherent biases in the antibodies, or to confounding biological noise. For each analyte in a protein measurement study, it is thus important to evaluate the technical and biological variation to account for confounding in the analytical signal. Our findings therefore support the utility of replicates in studies to assess analyte variation.

Lastly, in addition to assessing the inherent variability within and between the different protein assays, we also evaluated whether a matched case and control sisters study design would yield more statistical power in uncovering significant early diagnostic biomarkers. We initially hypothesized that sisters of the breast cancer cases would serve as well-matched controls as they are naturally controlled for race/ethnicity and a large proportion of genetic background. Moreover, our choice to evaluate pre-diagnostic samples eliminates bias associated with sample collection as both case and control samples were collected under the same conditions without knowledge of cancer diagnosis at the time of collection.

However, the unbiased principal component analysis exhibited that even with our careful study design choices to minimize bias, samples showed relatively large individual variance along their two principal component axes in the antibody-based datasets. Samples from biological sisters did not generally appear to be more similar to each other than to other individual samples (Additional file 3: Figure S1B). Hence it was not surprising to discover that the case and control samples were not well separated in the two-dimensional space of components that captured a substantive amount of the overall variance (>90%) (Additional file 3: Figure S1A).

Henceforth, we focused our analysis efforts on identifying individual protein analytes with potential diagnostic power. While a small number of proteins showed statistically significant differences between cases and controls; the changes were small (less than two-fold) and the number of proteins showing differences was small (Fig. 6). This finding is not necessarily unexpected. Given that the samples were collected from women up to 2 years before they were diagnosed with breast cancer, it is highly likely that the analytical signal in the blood plasma associated with breast cancer cannot be detected above the biological noise that arises from inter-individual variation.

We observed in Fig. 6a that the paired statistical analysis in the Olink platform provided more significant analytes than the unpaired analysis, suggesting that for the Olink platform, the paired design had greater statistical power when evaluating each protein analyte independently. However, also shown in Fig. 6a, we observed that there were some significant analytes that were mutually exclusive to the paired and unpaired analysis in the Myriad-RBM platform. If evaluating this platform in isolation, these results echo our initial PCA (Additional file 3: Figure S1); it appears that on the Myriad-RBM platform, the sisters are not more similar to each other than across all the samples both when protein levels are analyzed independently, and in covariance. This result may be explained by factors including, but not limited to, noise introduced by technical variation that was not evaluated for this platform, coupled with person-to-person variation from the samples. The Myriad-RBM oncology panel was chosen specifically for this study, and our results suggest that these protein measurements show little utility in pre-diagnostic breast cancer samples. This suggests that there is still a need for the unbiased exploratory LC-MS/MS methods that yield novel significant biomarker candidates as seen in Fig. 6b. Although the protein analytes in Fig. 6b have not been validated, they serve as a proof of concept that an untargeted approach can yield candidates that would otherwise be missed by targeted panels applied to noisy clinical samples.

## Conclusions

This study has comprehensively evaluated technical, practical and study design considerations for potential protein biomarker detection using the currently available technologies. Our findings provide a framework for larger studies and estimates of biological effect sizes for a more informed early protein cancer biomarker detection approach.

## Additional files

Additional file 1: Table S1. Subject characteristics of blood plasma samples from the Northern California site of the Breast Cancer Family Registry. **Table S2** Targeted proteins in the antibody-based proteomics platforms. (DOCX 25 kb)

Additional file 2: Measured Protein Levels Across LC-MS/MS, Olink, and Myriad-RBM platforms. (XLSX 98 kb)

Additional file 3: Figure S1. Principal Component Analysis. (a) first (*x*-axis) and second (*y*-axis) principal components capture more than 90% of variance in both Myriad-RBM and Olink datasets but are unable to separate case and control samples (supported visually by heavily overlapping red and blue density maps). (b) the same PCA plots as (a) with samples colored with anonymized sister pair indices reveal that, when evaluated in covariance, protein levels are not more similar between biological sisters than between unrelated individuals in the antibody-based assays. (DOCX 604 kb)

## Abbreviations

BCFR: Breast Cancer Family Registry
FABP4: Fatty Acid Binding Protein 4
IgA: Immunoglobulin A
IgG: Immunoglobulin G
LC-MS/MS: Liquid chromatography-tandem mass spectrometry
LDD: Least detectable concentration
PCA: Principal component analysis
qPCR: quantitative polymerase chain reaction
