# Supplementary material for: Assessing biological and technological variability in protein levels measured in pre-diagnostic plasma samples of women with breast cancer
Source: Biomark Res. 2017 Oct 17;5:30. doi: 10.1186/s40364-017-0110-y (PMC5645980; doi:10.1186/s40364-017-0110-y)
Supplement: Supplementary file 1 — Subject characteristics of blood plasma samples from the Northern California site of the Breast Cancer Family Registry. Table S2 Targeted proteins in the antibody-based proteomics platforms. (DOCX 25 kb) [file 40364_2017_110_MOESM1_ESM.docx]

# SUPPLEMENTAL MATERIALS

**Supplemental Table S1:** Subject characteristics of blood plasma samples from the Northern California site of the Breast Cancer Family Registry

| Race/ethnicity | Age at Blood Draw (years) | Age at Diagnosis (years) | Menopausal Status at blood draw | Age of Sister at Blood Draw (years) | Age of Sister at Follow-Up (years) |
| --- | --- | --- | --- | --- | --- |
| Asian American | 40 | 40 | Pre | 43 | 61 |
| Hispanic | 57 | 57 | Post | 61 | 76 |
| Asian American | 44 | 44 | Pre | 42 | 60 |
| African American | 85 | 85 | Post | 78 | 86 |
| Hispanic | 55 | 55 | Post | 57 | 70 |
| Hispanic | 57 | 57 | Post | 53 | 62 |
| Non-Hispanic White | 41 | 42 | Pre | 48 | 67 |
| Hispanic | 42 | 43 | Pre | 56 | 65 |
| Non-Hispanic White | 49 | 51 | Post | 44 | 60 |
| Hispanic | 52 | 55 | Post | 40 | 52 |

**Supplemental Table S2:** Targeted proteins in the antibody-based proteomics platforms

| Protein Name | Gene Symbol | UniProt Accession | Myriad-RBM | Olink |
| --- | --- | --- | --- | --- |
| Tartrate-resistant acid phosphatase type 5 | ACP5 | P13686 |  | x |
| Proadrenomedullin N-20 terminal peptide | ADM | P35318 |  | x |
| Alpha-Fetoprotein | AFP | P02771 | x |  |
| Aldose Reductase | AKR1B1 | P15121 | x |  |
| Angiogenin | ANG | P03950 | x |  |
| Annexin A1 | ANXA1 | P04083 | x |  |
| Amphiregulin | AREG | P15514 | x | x |
| Bcl-2-like protein 2 | BCL2L2 | Q92843 | x |  |
| Basigin | BSG | P35613 |  | x |
| Betacellulin | BTC | P35070 | x | x |
| CA242 | CA242 | CA242 |  | x |
| Cancer Antigen 72-4 | CA724 | CA724 | x |  |
| Carbonic anhydrase 9 | CA9 | Q16790 |  | x |
| Caspase-3 | CASP3 | P42574 |  | x |
| C-C motif chemokine 19 | CCL19 | Q99731 |  | x |
| Monocyte Chemotactic Protein 1 | CCL2 | P13500 | x | x |
| C-C motif chemokine 21 | CCL21 | O00585 | x | x |
| Macrophage inflammatory protein 3 beta | CCL23 | P55773 | x |  |
| Eotaxin-2 | CCL24 | O00175 | x | x |
| CD40 ligand, membrane form | CD40LG | P29965 |  | x |
| Early activation antigen CD69 | CD69 | Q07108 |  | x |
| Carcinoembryonic Antigen | CEACAM5 | P06731 | x | x |
| Human Chorionic Gonadotropin beta | CGB | P01233 | x |  |
| Chitinase-3-like protein 1 | CHI3L1 | P36222 | x |  |
| Tetranectin | CLEC3B | P05452 | x |  |
| Endostatin | COL18A1 | P39060 | x |  |
| Collagen IV | COL4A4 | P53420 | x |  |
| Processed macrophage colony-stimulating factor 1 | CSF1 | P09603 |  | x |
| Granulocyte-macrophage colony-stimulating factor | CSF2 | P04141 |  | x |
| Cystatin-B | CSTB | P04080 |  | x |
| Cathepsin D | CTSD | P07339 | x | x |
| Interferon gamma Induced Protein 10 | CXCL10 | P02778 | x | x |
| Interferon-inducible T-cell alpha chemoattractant | CXCL11 | O14625 | x | x |
| Stromal cell-derived factor-1 | CXCL12 | P48061 | x |  |
| B Lymphocyte Chemoattractant | CXCL13 | O43927 | x | x |
| C-X-C motif chemokine 5 | CXCL5 | P42830 |  | x |
| Interleukin-8 | CXCL8 | P10145 |  | x |
| Monokine Induced by Gamma Interferon | CXCL9 | Q07325 | x | x |
| Epidermal Growth Factor | EGF | P01133 | x | x |
| Epidermal Growth Factor Receptor | EGFR | P00533 | x | x |
| Endoglin | ENG | P17813 | x |  |
| Neuron-Specific Enolase | ENO2 | P09104 | x |  |
| Epithelial cell adhesion molecule | EPCAM | P16422 | x | x |
| Erythropoietin | EPO | P01588 |  | x |
| Human Epidermal Growth Factor Receptor 2 | ERBB2 | P04626 | x | x |
| Receptor tyrosine-protein kinase erbB-3 | ERBB3 | P21860 | x | x |
| Receptor tyrosine-protein kinase erbB-4 | ERBB4 | Q15303 |  | x |
| Epiregulin | EREG | O14944 | x | x |
| Estrogen receptor | ESR1 | P03372 |  | x |
| Ezrin | EZR | P15311 | x |  |
| Tissue factor | F3 | P13726 |  | x |
| Fatty Acid-Binding Protein, liver | FABP1 | P07148 | x |  |
| Fatty Acid-Binding Protein, adipocyte | FABP4 | P15090 | x | x |
| Tumor necrosis factor receptor superfamily member 6 | FAS | P25445 |  | x |
| ADAM10-processed FasL form | FASLG | P48023 |  | x |
| Fibulin-1C | FBLN1 | P23142 | x |  |
| Fibroblast Growth Factor basic | FGF1 | P05230 | x |  |
| Vascular endothelial growth factor D | FIGF | O43915 | x | x |
| Vascular Endothelial Growth Factor Receptor 1 | FLT1 | P17948 | x |  |
| Fms-related tyrosine kinase 3 ligand | FLT3LG | P49771 |  | x |
| Vascular endothelial growth factor receptor 3 | FLT4 | P35916 | x |  |
| Cellular Fibronectin | FN1 | P02751 | x |  |
| Folate receptor alpha | FOLR1 | P15328 |  | x |
| Follistatin | FST | P19883 |  | x |
| Glutamate-Cysteine Ligase Regulatory subunit | GCLM | P48507 | x |  |
| Growth/differentiation factor 15 | GDF15 | Q99988 |  | x |
| Somatotropin | GH1 | P01241 |  | x |
| Lactoylglutathione lyase | GLO1 | Q04760 | x |  |
| Glucose-6-phosphate Isomerase | GPI | P06744 | x |  |
| Gelsolin | GSN | P06396 | x |  |
| Glutathione S-Transferase Mu 1 | GSTM1 | P09488 | x |  |
| Heparin-Binding EGF-Like Growth Factor | HBEGF | Q99075 | x | x |
| Hepatocyte Growth Factor | HGF | P14210 | x | x |
| Hepsin | HPN | P05981 | x |  |
| Interferon gamma | IFNG | P01579 |  | x |
| Insulin-like Growth Factor-Binding Protein 1 | IGFBP1 | P08833 | x |  |
| Insulin-like Growth Factor-Binding Protein 2 | IGFBP2 | P18065 | x |  |
| Insulin-like Growth Factor-Binding Protein 3 | IGFBP3 | P17936 | x |  |
| Insulin-like Growth Factor Binding Protein 4 | IGFBP4 | P22692 | x |  |
| Insulin-like Growth Factor Binding Protein 5 | IGFBP5 | P24593 | x |  |
| Insulin-like Growth Factor Binding Protein 6 | IGFBP6 | P24592 | x |  |
| Interleukin-12 subunit beta | IL12B | P29460 |  | x |
| Interleukin-17 receptor B | IL17RB | Q9NRM6 |  | x |
| Interleukin-1 receptor antagonist protein | IL1RN | P18510 |  | x |
| Interleukin-2 | IL2 | P60568 |  | x |
| Interleukin-2 receptor alpha | IL2RA | P01589 | x | x |
| Interleukin-4 | IL4 | P05112 |  | x |
| Interleukin-6 | IL6 | P05231 | x | x |
| Interleukin-6 receptor subunit alpha | IL6R | P08887 |  | x |
| Interleukin-6 receptor subunit beta | IL6ST | P40189 | x |  |
| Interleukin-7 | IL7 | P13232 |  | x |
| Vascular Endothelial Growth Factor Receptor 2 | KDR | P35968 | x | x |
| Kit ligand | KITLG | P21583 |  | x |
| Kallikrein-11 inactive chain 1 | KLK11 | Q9UBX7 |  | x |
| Prostate-specific antigen | KLK3 | P07288 |  | x |
| Kallikrein 5 | KLK5 | Q9Y337 | x |  |
| Kallikrein-6 | KLK6 | Q92876 |  | x |
| Kallikrein-7 | KLK7 | P49862 | x |  |
| Neutrophil Gelatinase-Associated Lipocalin | LCN2 | P80188 | x |  |
| Leptin | LEP | P41159 | x |  |
| Galectin-3 | LGALS3 | P17931 | x | x |
| Midkine | MDK | P21741 |  | x |
| Hepatocyte Growth Factor receptor | MET | P08581 | x | x |
| Melanoma-derived growth regulatory protein | MIA | Q16674 |  | x |
| MHC class I chain-related protein A | MICA | Q29983 | x | x |
| Macrophage Migration Inhibitory Factor | MIF | P14174 | x |  |
| Matrix Metalloproteinase-2 | MMP2 | P08253 | x |  |
| Stromelysin-1 | MMP3 | P08254 |  | x |
| Myeloperoxidase | MPO | P05164 |  | x |
| Mesothelin | MSLN | Q13421 | x |  |
| Macrophage-Stimulating Protein | MST1 | P26927 | x |  |
| Cancer Antigen 15-3 | MUC1 | P15941 | x |  |
| Cancer Antigen 125 | MUC16 | Q8WXI7 | x | x |
| Myeloid differentiation primary response protein MyD88 | MYD88 | Q99836 |  | x |
| Nucleoside diphosphate kinase B | NME2 | P22392 | x |  |
| Neuropilin-1 | NRP1 | O14786 | x |  |
| Platelet-Derived Growth Factor BB | PDGFB | P01127 | x | x |
| Platelet endothelial cell adhesion molecule | PECAM1 | P16284 |  | x |
| Pepsinogen I | PG1 | O95576 | x |  |
| Placenta Growth Factor | PGF | P49763 | x |  |
| Tissue type Plasminogen activator | PLAT | P00750 | x |  |
| Urokinase-type plasminogen activator receptor | PLAUR | Q03405 | x | x |
| Placenta growth factor | PLGF | P49763 |  | x |
| Urokinase-type Plasminogen Activator | PRAP1 | Q96NZ9 | x |  |
| Peroxiredoxin-4 | PRDX4 | Q13162 | x |  |
| Prolactin | PRL | P01236 |  | x |
| Prostasin | PRSS8 | Q16651 | x | x |
| Phosphoserine Aminotransferase | PSAT1 | Q9Y617 | x |  |
| Regenerating islet-derived protein 4 | REG4 | Q9BYZ8 |  | x |
| Protein S100-A4 | S100A4 | P26447 | x |  |
| Protein S100-A6 | S100A6 | P06703 | x |  |
| E-selectin | SELE | P16581 |  | x |
| Squamous Cell Carcinoma Antigen-1 | SERPINB3 | P29508 | x |  |
| Maspin | SERPINB5 | P36952 | x |  |
| Osteopontin | SPP1 | P10451 | x |  |
| Cancer Antigen 19-9 | ST6GALNAC6 | Q969X2 | x |  |
| Tyrosine kinase with Ig and EGF homology domains 2 | TEK | Q02763 | x | x |
| Thyroglobulin | TG | P01266 | x |  |
| Transforming Growth Factor alpha | TGFA | P01135 | x | x |
| Latency-Associated Peptide of Transforming Growth Factor beta 1 | TGFB1 | P01137 | x | x |
| Thrombopoietin | THPO | P40225 |  | x |
| Tenascin-C | TNC | P24821 | x |  |
| Tumor necrosis factor, membrane form | TNF | P01375 |  | x |
| Osteoprotegerin | TNFRSF11B | O00300 | x | x |
| Tumor Necrosis Factor Receptor I | TNFRSF1A | P19438 | x | x |
| Tumor necrosis factor receptor superfamily member 1b | TNFRSF1B | P20333 |  | x |
| Tumor necrosis factor receptor superfamily member 4 | TNFRSF4 | P43489 |  | x |
| B cell-activating factor | TNFSF13B | Q9Y275 | x | x |
| Tumor necrosis factor ligand superfamily member 14 | TNFSF14 | O43557 |  | x |
| Tumor necrosis factor ligand superfamily member 8 | TNFSF8 | P32971 |  | x |
| Vascular Endothelial Growth Factor | VEGFA | P15692 | x | x |
| Vascular endothelial growth factor B | VEGFB | P49765 | x |  |
| Vascular Endothelial Growth Factor C | VEGFC | P49767 | x |  |
| WAP four-disulfide core domain protein 2 | WFDC2 | Q14508 | x | x |
