# Supplementary material for: Assessing biological and technological variability in protein levels measured in pre-diagnostic plasma samples of women with breast cancer
Source: Biomark Res. 2017 Oct 17;5:30. doi: 10.1186/s40364-017-0110-y (PMC5645980; doi:10.1186/s40364-017-0110-y)
Supplement: Supplementary file 3 — Principal Component Analysis. (a) first (x-axis) and second (y-axis) principal components capture more than 90% of variance in both Myriad-RBM and Olink datasets but are unable to separate case and control samples (supported visually by heavily overlapping red and blue density maps). (b) the same PCA plots as (a) with samples colored with anonymized sister pair indices reveal that, when evaluated in covariance, protein levels are not more similar between biological sisters than between unrelated individuals in the antibody-based assays. (DOCX 604 kb) [file 40364_2017_110_MOESM3_ESM.docx]

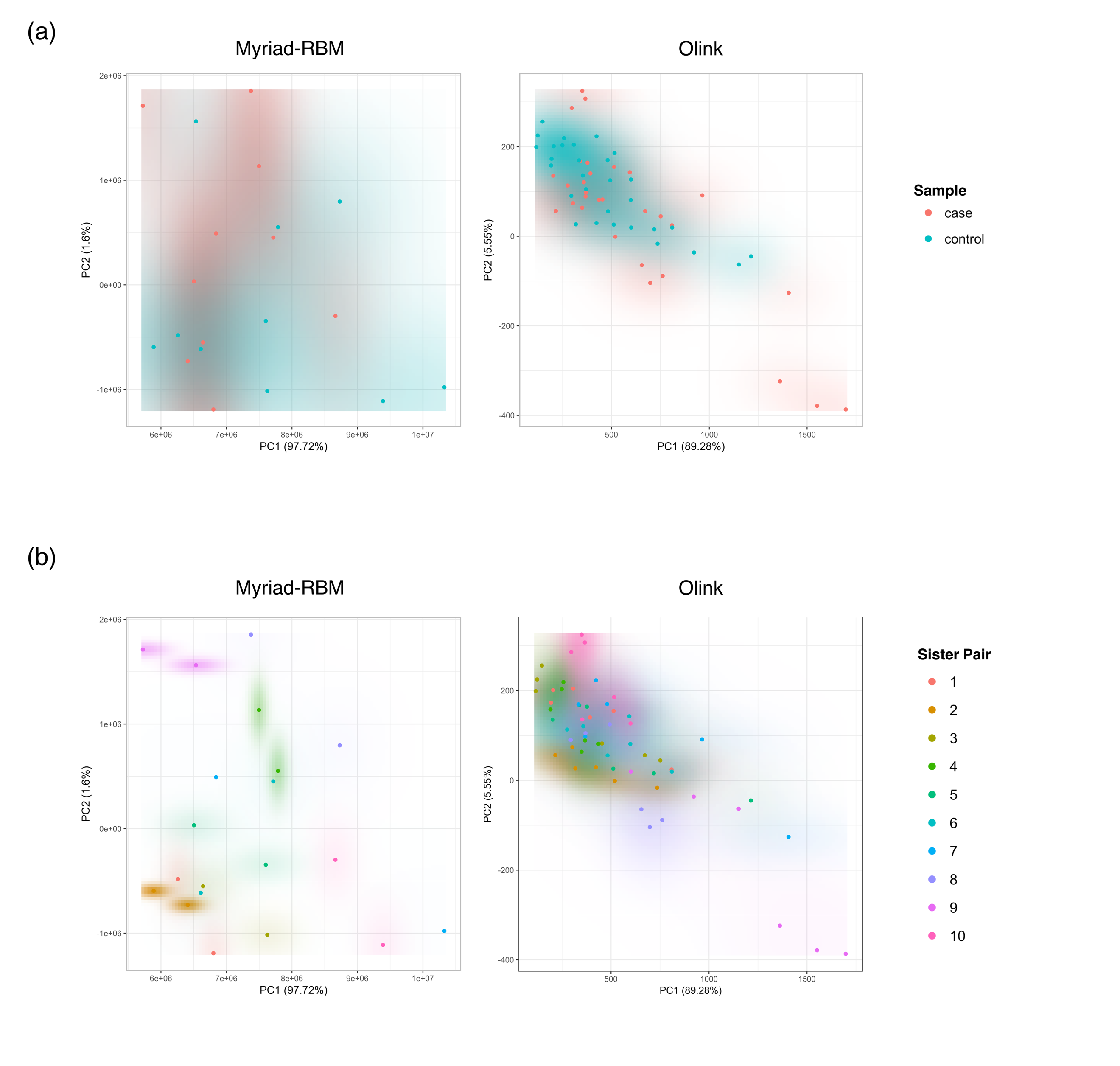


**Figure S1: Principal Component Analysis.** (a) first (*x*-axis) and second (*y*-axis) principal components capture more than 90% of variance in both Myriad-RBM and Olink datasets but are unable to separate case and control samples (supported visually by heavily overlapping red and blue density maps). (b) the same PCA plots as (a) with samples colored with anonymized sister pair indices reveal that, when evaluated in covariance, protein levels are not more similar between biological sisters than between unrelated individuals in the antibody-based assays.
